# Magnesium—A Potential Key Player in Inflammatory Bowel Diseases?

**DOI:** 10.3390/nu14091914

**Published:** 2022-05-03

**Authors:** Georgiana-Emmanuela Gilca-Blanariu, Anca Trifan, Manuela Ciocoiu, Iolanda Valentina Popa, Alexandru Burlacu, Gheorghe G. Balan, Andrei Vasile Olteanu, Gabriela Stefanescu

**Affiliations:** 1 Faculty of Medicine, “Grigore T. Popa” University of Medicine and Pharmacy, 700115 Iasi, Romania; georgiana.gilca@gmail.com (G.-E.G.-B.); ancatrifan@yahoo.com (A.T.); mciocoiu2003@yahoo.com (M.C.); balan.gheo@yahoo.com (G.G.B.); olteanuandrei@yahoo.com (A.V.O.); gabriela.stefanescu@gmail.com (G.S.); 2 Department of Gastroenterology and Hepatology, “Sf. Spiridon” County Clinical Emergency Hospital, 700111 Iasi, Romania; 3 Department of Interventional Cardiology, Cardiovascular Diseases Institute, 700503 Iasi, Romania

**Keywords:** inflammatory bowel disease, magnesium, depression, anxiety, sleep, insomnia, sleep quality, PSQI, HADS

## Abstract

The altered magnesium status in inflammatory bowel disease (IBD) patients may have a significant clinical imprint considering its role in cell signaling and genomic stability, as well as its involvement in IBD patients’ fatigue. Our study pioneers the investigation of magnesium hair concentration patterns in an adult population of IBD patients. The hair magnesium concentration in IBD patients is compared to healthy controls in order to identify correlations between the magnesium status and relevant parameters related to disease activity, psychological status, and sleep quality. We report a significantly lower hair magnesium concentration within the IBD group compared to healthy controls (95%CI: 0.006–0.062; *p* = 0.017) and lower levels in CD compared to UC (95%CI: −0.061–−0.002; *p* = 0.038). We identified a borderline statistical significance between the hair magnesium concentration and UC disease activity (95%CI; −0.679–0.008; *p* = 0.055) and a significantly lower magnesium concentration in patients who reported increased sleep latency (95%CI −0.65–−0.102; *p* = 0.011) or decreased sleep duration (95%CI −0.613–−0.041; *p* = 0.028). Our results advance several hypotheses with substantial clinical impact to be confirmed in future studies. Magnesium levels appear to be modified in IBD patients, which suggests it either plays a primary role in disease pathophysiology or a is result of the disease’s evolution. Magnesium could be used in predictive models for clinical/subclinical disease activity. Moreover, magnesium supplementation may improve IBD evolution and sleep quality for patients with a deficit of this mineral. However, confirmatory evidence-based studies are needed to generate specific dosing, time of supplementation, and optimum monitoring of magnesium status in IBD patients.

## 1. Introduction

Altered nutritional status among inflammatory bowel disease (IBD) patients appears to be frequent, considering existing data, with an estimated prevalence of malnutrition reaching 65–75% among patients with Crohn’s disease (CD) [1] and 18–62% among ulcerative colitis (UC) patients [2,3]. Malnutrition can involve protein-calorie malnutrition and micronutrient deficiency, thereby influencing the disease’s course and leading to poorer outcomes, including prolonged hospital stay [4], complicated perioperative course, and even higher mortality [5].

There is recent evidence that magnesium contributes to regulating cellular timekeeping in animal cells [6], and, consequently, has a beneficial effect on maintaining normal circadian rhythm and contributes to improved quality of sleep in humans [7]. Moreover, low dietary magnesium intake was significantly associated with depression [8]; it is known that depression is associated with poor sleep [8].

Despite the real impact of magnesium, the micronutrient’s deficiency can be easily overlooked considering the absence of specific clinical signs. Moreover, selecting the optimal sampling method to detect magnesium concentration is challenging in the context of IBD. Micronutrient deficiency can be multifactorial, and inflammation can affect serum micronutrient concentrations. Consequently, magnesium serum levels can have a limited value in reflecting the status of this trace element in chronic inflammatory diseases [9]. For this reason, we opted for assessing magnesium status in hair samples.

We aimed to investigate the particularities and patterns of hair magnesium concentration in IBD patients compared to healthy controls and investigate the presence of correlations between magnesium levels and disease activity, sleep, and psychological impairment among IBD patients.

## 2. Materials and Methods

We have conducted an ancillary study derived from a single prospective center case-control study in which we evaluated the mineral and trace elements status among patients with inflammatory bowel disease (IBD) by measuring hair concentration using Scanning Electron Microscope (SEM) and Energy Dispersive X-ray spectroscopy (EDX), and we also investigated the relationship between the micronutrients’ status, disease activity, and systemic inflammatory markers [10].

### 2.1. Subjects

The research group was comprised of individuals who had a confirmed diagnosis of IBD based on clinical presentation, endoscopic and histological results, and were between the ages of 18 and 70 years. Patients with concurrent infections; those taking micronutrient supplements; and those with a history of neoplasia, metabolic disorders (obesity, dyslipidemia, and thyroid dysfunction), or diseases suspected to be magnesium-related (attention deficit/hyperactivity disorder, autism spectrum disorder, Parkinson’s disease, or cardiovascular disease) were excluded. The Crohn’s disease activity index (CDAI) was used to assess disease activity in CD patients, while the clinical Mayo score was used to assess disease activity in UC patients. The control group included healthy people (*n* = 37) who had undergone a standard clinical examination and had no history of chronic illnesses, gastrointestinal problems, or autoimmune diseases. The exclusion criteria for both the research and the control group were the use of hair therapeutic agents or cosmetics (medicinal shampoos that may interfere with the outcomes of the studies) and restricted diets within the preceding six months. In the study and control groups, magnesium hair concentration, serum albumin, and C reactive protein (CRP) levels were determined. CRP and albumin were included in our analysis on the basis of their proven prognostic and predictive power in IBD, as acknowledged by several consensus guidelines and studies [11,12,13,14]. As chronic inflammation increases albumin catabolism [13], it is possible to utilize low albumin levels as a surrogate marker for severe inflammation and extensive colitis [15], as well as to predict the need for colectomy in IBD [16]. Psychological status (Hospital Anxiety and Depression Score (HADS) for anxiety and depression) and also sleep quality (using Pittsburgh Sleep Quality Index (PSQI) were also evaluated within the study group.

HADS is a frequently used and validated tool for assessing anxiety and depression in various clinical settings. This questionnaire includes seven items for evaluating anxiety and seven items for evaluating depression. A score below 7 is considered normal, whereas a score of 8 to 10 is considered borderline abnormal, while a score of 10 or higher is framed as abnormal. In the performed study, we considered a score above eight abnormal. We used a binary transformation for HADS, defining a HADS value lower than 8 as standard and higher than 8 as altered.

As a widely used instrument for assessing sleep quality in both research and clinical settings [17], the PSQI evaluates seven components, which evoke different types of events over the last month. The following items pertain to seven different aspects of sleep quality: subjective sleep quality evaluated by the patient, use of sleep medication, sleep latency, sleep duration, habitual sleep efficiency, and the degree of daytime dysfunction resulting from inadequate sleep. The patient response scores between 0—representing the absence of deterioration of the component—and 3—a significant alteration for each of the seven components assessed. As a result, the final score might range anywhere from 0 to 21. A result higher than or equal to 5 corresponds to poor sleep quality [18,19]. We used a binary transformation for each PSQI component: PSQI components with a value of 0 are categorized as standard and PSQI components with values higher than one are considered altered.

### 2.2. Hair Sample Collection and SEM and EDX Measurement

According to the most recent hair analysis standards [20], the hair sample was obtained from the occipital area and comprised a minimum of 10 strands for each subject. The EDX studies were carried out on the root end of the samples after they had been cleaned using the Hess technique [21]. The hair strands were placed in tiny plates with distilled water containing a drop of detergent and were sonicated for 5 min. After that, the sample was rinsed in distilled water, sonicated in 100% acetone for 5 min, and allowed to dry.

After washing, the hair samples were analyzed using a Quanta 200 Scanning Electron Microscope (SEM, FEI Company, Hillsboro, OR, USA) in high vacuum mode at 30 kV. For element identification and quantitative analysis, the EDX system (FEI Company, USA) placed on the Quanta 200 SEM was employed. The surface and cross-section of the hair specimens were elementally analyzed. There was no need for any additional treatment or processing.

### 2.3. Ethical Considerations

The blood and hair samples were handled in accordance with European Directive EC No 206, and the Scientific Council accepted the technique employed at the Petru Poni Institute of Macromolecular Chemistry for processing the hair samples undergoing EDX examination. The study protocol and all procedures included in the study adhered to the Declaration of Helsinki’s ethical standards and were approved by the Ethical Committee of “Grigore T. Popa” University of Medicine and Pharmacy (25 November 2018), “Sf. Spiridon” County Clinical Emergency Hospital (No 45/04.09/2019). All patients included in the trial provided signed informed consent upon enrollment.

### 2.4. Statistical Analysis

The data were analyzed by R Studio Version 1.2.1335 © 2009–2019 Rstudio (Inc. Build 1379, Boston, MA, USA). The continuous variables were presented as a mean (±standard deviation). Means of two normally-distributed continuous variables were compared by independent samples using a Student’s *t*-test. In the case of non-normal distributions, continuous variables were compared using the Kruskall–Wallis test. The correlation between two continuous variables was studied using Spearman’s rank correlation. The frequencies of the categorical variables were expressed as absolute values and percentages and were compared using Pearson χ2. The point–biserial correlation assessed the association between a continuous variable and a categorical one. A logistic regression model was implemented for the prediction of IBD activity based on several variables (such as magnesium, CRP, serum albumin level). The performance of the predictive models was assessed by several metrics, such as the area under the receiver operating characteristic curve (AUC), accuracy, sensitivity, and specificity. A value of *p* < 0.05 was considered significant.

## 3. Results

### 3.1. Patients Included

Thirty-seven patients with IBD (25 with UC and 12 with CD) and 31 control volunteers were included in our study. Table 1 summarizes the baseline demographic and clinical data.

### 3.2. Magnesium Deficiency in IBD and Subtypes

**Figure 1:**
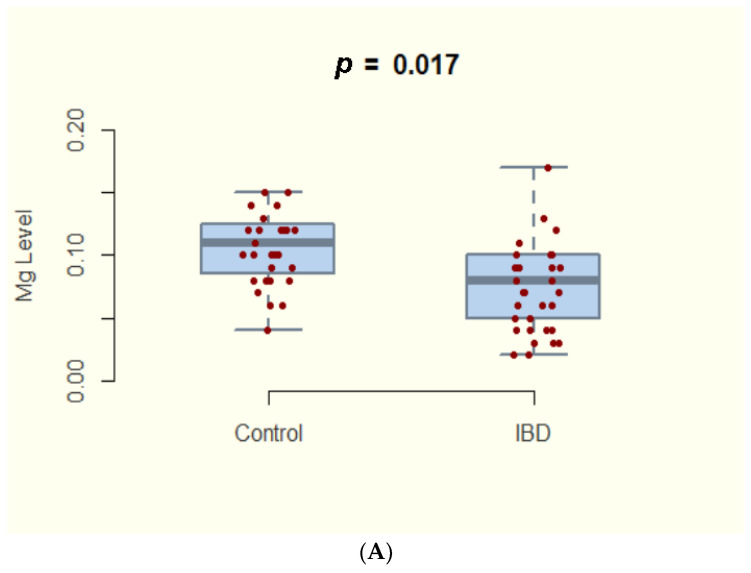

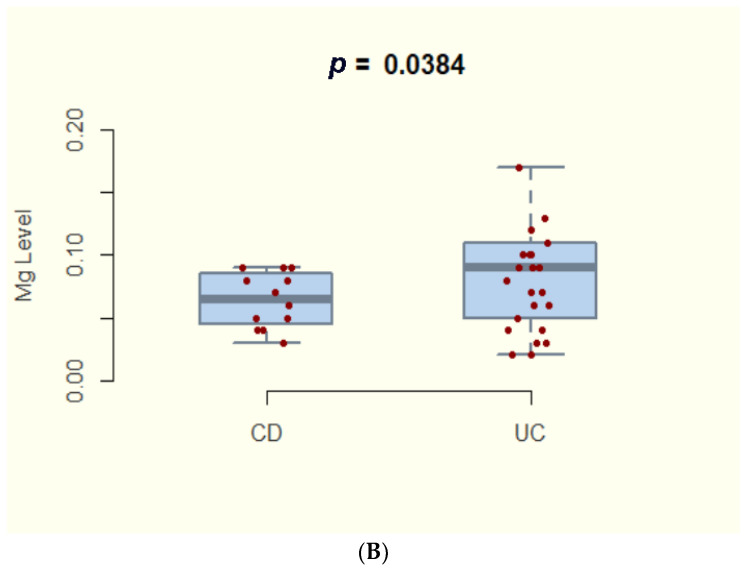
(**A**) Evaluating the difference in hair magnesium concentration between IBD (inflammatory bowel disease) patients and the control group. (**B**) Evaluating the difference in hair magnesium concentration between CD (crohn’s disease)and UC (ulcerative colitis).

We report a statistically significant lower hair magnesium concentration within the IBD group than in healthy controls (95%CI: 0.006–0.062; *p* = 0.017) (Figure 1A).

Considering the magnesium deficit identified within the IBD group, we studied the presence of significant differences between IBD subtypes achieving significantly lower concentrations of magnesium in CD compared to UC (95%CI: −0.061–−0.002; *p* = 0.038) (Figure 1B).

### 3.3. Magnesium Deficiency and Disease Activity

We have also evaluated the correlation between magnesium concentration and disease activity in CD patients without highlighting any significant associations (95%CI; −0.649–0.488; *p* = 0.713).

**Figure 2:**
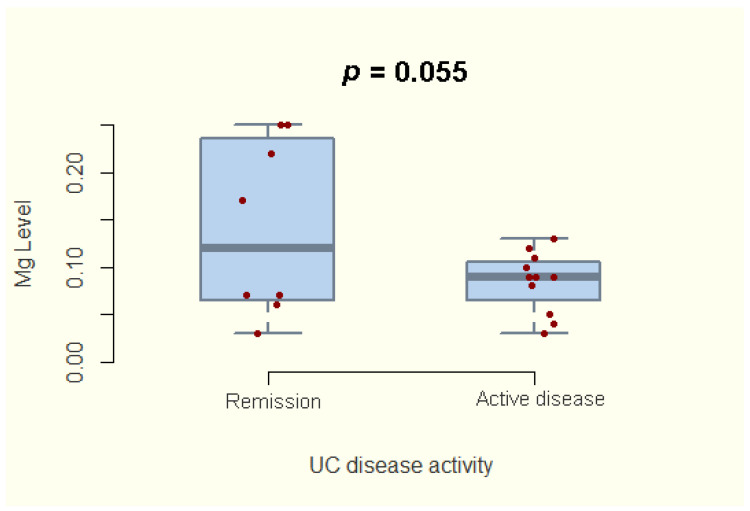
Evaluating the difference in hair magnesium concentration for UC patients based on disease activity.

As far as the correlation between magnesium status and UC activity, we identified a borderline statistical significance between hair magnesium concentration and disease activity (95%CI; −0.679–0.008; *p* = 0.055) (Figure 2).

### 3.4. Integrating Magnesium Concentration in a Predictive Model for UC Disease Activity

**Figure 3:**
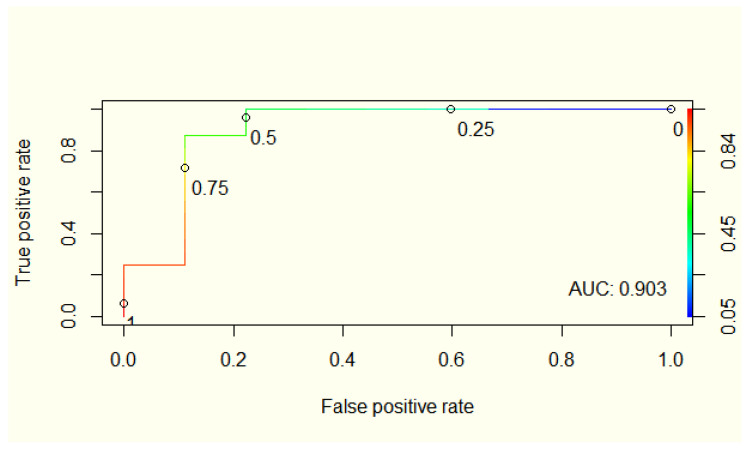
Logistic regression model for predicting UC disease activity based on magnesium, CRP (C reactive protein), and serum albumin level.

Considering the borderline statistical significance between hair magnesium concentration and UC disease activity, we developed a logistic regression model for predicting UC disease activity based on magnesium, CRP, and serum albumin level, which achieved a good performance (AUC—0.9, accuracy—0.88, sensitivity—0.78, specificity—0.94) (Figure 3). Adding magnesium improves the predictive model’s performance based only on CRP and serum albumin levels (AUC—0.85, accuracy—0.8, sensitivity—0.67, specificity—0.88).

### 3.5. Nutritional Status and Magnesium Hair Concentration

When assessing the presence of correlations between magnesium concentration and nutritional status, we have not found any significant association—neither for the UC group (BMI, *p* = 0.653; serum albumin, *p* = 0.369) nor for the CD patients (BMI, *p* = 0.759; serum albumin: *p* = 013).

### 3.6. Psychological Status and Magnesium Hair Concentration

We evaluated the relationship between magnesium concentration and psychological distress among IBD patients. We did not find significant associations between the micronutrient’s status and anxiety, which was reflected through the HADS-A score (95%CI −0.278–0.381; *p* = 0.727), nor the micronutrient’s status and depression, which was reflected through HADS-D (95%CI −0.409–0.243; *p* = 0.59).

### 3.7. Sleep Quality and Magnesium Hair Concentration

**Figure 4:**
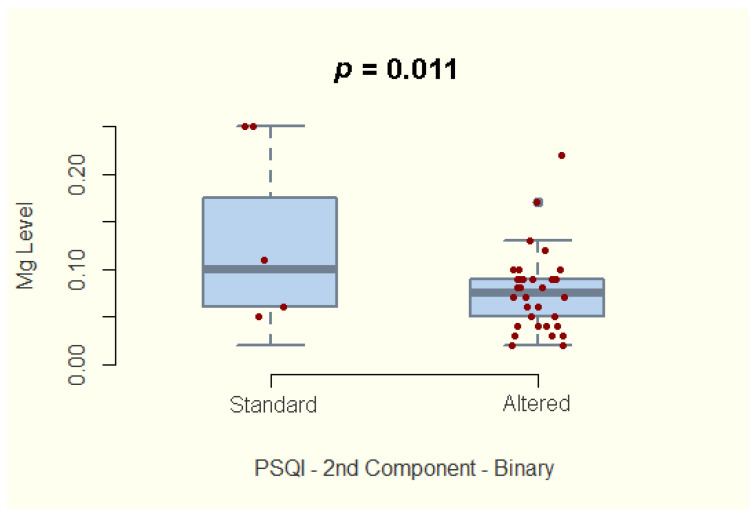
Evaluating the correlation between hair magnesium concentration and sleep latency (component 2 of the PSQI score) among IBD patients.

**Figure 5:**
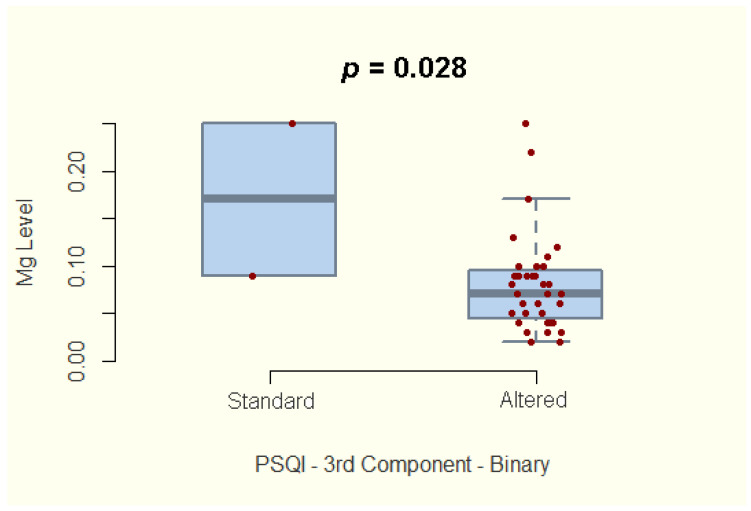
Evaluating the correlation between hair magnesium concentration and sleep duration (component 3 of the PSQI score) among IBD patients. PSQI: Pittsburgh Sleep Quality Index.

A relationship between the magnesium concentration and the quality of sleep among IBD patients was also explored. We report no significant association with the global PSQI score. However, our study shows a significantly lower magnesium concentration in patients who reported increased sleep latency (component 2 of the PSQI score, 95%CI −0.65–−0.102; *p* = 0.011) or decreased sleep duration (component 3 of the PSQI score, 95%CI −0.613–−0.041; *p* = 0.028) (Figure 4; Figure 5).

Table 2 illustrates the statistical significance of the correlations between magnesium hair concentration and each PSQI subcomponent.

## 4. Discussion

Our study is the first to assess magnesium hair concentration patterns in an adult IBD population, and therefore introduces a new, original perspective for the evaluation of IBD patients. Only one published study evaluated magnesium hair levels in IBD, but in a pediatric population [9].

Several contributing processes can lead to micronutrient deficiencies in IBD patients [3]. Inadequate dietary intake and chronic loss from diarrhea can lead to magnesium depletion. The altered magnesium status has a significant clinical imprint, due to its role in cell signaling, genome integrity, and DNA repair systems [22,23,24]—and can also be involved in IBD patients’ fatigue [25]. However, magnesium deficiency in IBD is still insufficiently studied, and its importance could be undermined in the pathophysiology of IBD.

Considering the importance of identifying magnesium deficits in IBD, it is essential to choose the optimal sampling method to detect magnesium levels. The most frequently used method is measuring serum concentration, an accessible option [25,26]. However, because inflammation has been shown to affect blood micronutrient concentrations, serum levels in chronic inflammatory illnesses like IBD may be ineffective in representing global micronutrient status [9,27,28]. Moreover, serum magnesium levels are not necessarily indicative of total body magnesium status [29]. As a result, the widespread magnesium deficit may be overshadowed by normal blood magnesium levels [29]. Hair deposits may provide more precise reflections of total body magnesium status, thereby allowing for better detection of magnesium-deficient people and the prevention of magnesium-related complications [30]. Consequently, we opted for evaluating magnesium status by measuring the hair concentration. This approach has been used in various diseases [31], ranging from diabetes [32] to autoimmune disorders, with evidence for its applicability in patients with psoriasis [33] and alopecia areata [34].

Our study identified magnesium deficiency among IBD patients compared to healthy controls. In contrast, Cho et al. [9] did not identify significant differences between IBD and healthy children regarding magnesium hair levels. It remains to be further studied whether this difference in results is generalizable and can be explained by the particular physiology of IBD children compared to adults.

Furthermore, we identified a statistically significant lower hair magnesium concentration among CD patients compared to UC patients, which might be due to the ileal involvement in CD patients, thereby leading to impaired absorption. Again, no difference between UC and CD was observed by Cho et al. in the pediatric population [9]. More studies, both in adults and children, should further replicate the results to draw firm evidence-based conclusions.

Another element of novelty in our study is the inclusion of magnesium levels in a predictive model to estimate the disease activity in UC. A score that monitors disease activity aims to detect early signs of relapse for prompt intervention and treatment, thus preventing severe outbreaks and reducing the socio-economic impact [35]. Magnesium increased the predictive power of a disease activity model based on traditional predictors such as CRP and albumin. The advantage of this score is that it allows for estimating the disease activity in patients in which anamnesis is difficult or impossible.

We also investigated the presence of the correlations between magnesium concentration and altered psychological status, anxiety, and depression through HADS. Magnesium deficiency has an impact on psychological well-being in addition to the physical manifestations such as fatigue, muscular cramps and arrhythmia. Hypomagnesemia has previously been linked to depression in people with IBD [36].

There was no statistically significant association between low hair magnesium levels and either anxiety or depression scores in our IBD group. This could be due to the fact that hair magnesium concentration reflects the status of this micronutrient for a more extended period (about three months), while the psychological status evaluated through HADS mirrors the status within the last week. Therefore, the serum magnesium concentration would help complete the biological panel of investigations in this setting. The low number of IBD patients included in our study could also be involved in not reaching the statistical significance for the association between anxiety/depression scores and hair magnesium concentration.

Considering the documented influence of magnesium on sleep [37], we studied the correlation between hair magnesium concentration and quality of sleep measured by the PSQI score. We did not find statistically significant differences regarding hair magnesium concentration between IBD patients with impaired quality of sleep and IBD patients with average PSQI scores when comparing the global score. However, we identified statistically significant lower hair magnesium concentrations among patients who reported increased sleep latency and reduced sleep duration. Whether the magnesium supplementation would reduce sleep latency and increase sleep duration remains to be seen in future randomized clinical trials. Whether the intervention will have positive effects, IBD patients will benefit from another critical therapy for better disease evolution and quality of life.

Our study has several limitations. Firstly, the small number of patients limits the generalizability of the results. Secondly, magnesium serum concentrations were not documented, thereby hindering an insight into optimizing the magnesium status evaluation by using seric and hair concentrations, and therefore potentially offering a broader perspective of the whole-body magnesium status and its impact. Thirdly, no data related to the follow-up visits have been included in the current study; this aspect could be targeted by a future study, which could evaluate the potential role of magnesium through its relationship with disease evolution over time. Fourthly, no data regarding PSQI scores of the healthy controls was collected. In the future, direct comparisons between the PSQI scores of IBD patients and healthy controls in relation to magnesium could bring new and impactful insights.

## 5. Conclusions

Our study pioneers the investigation of magnesium hair concentration in an adult population of IBD patients. Our results advance several hypotheses with substantial clinical impacts to be confirmed in future studies. Magnesium levels appear to be modified in IBD patients, which suggests that either a primary role is played in disease pathophysiology or is a result of the disease evolution. Magnesium could be integrated in predictive models for disease activity. Considering the association between magnesium, disease activity, and sleep quality, which was identified in our study, further research may assess whether magnesium supplementation could improve IBD evolution and sleep quality in patients who are deficient in this mineral. Confirmatory evidence-based studies will be needed to establish appropriate dosing, timing, and monitoring of magnesium status in IBD patients.

## Figures and Tables

**Table 1:** Patient characteristics.

Patient Characteristics	Study Group *n* = 37		Control Group *n* = 31	*p*-Value
	UC (*n* = 25)	CD (*n* = 12)		
Age, median (Q25; Q75)	43.5 (30; 59.5)		32 (29; 42)	0.05 ^§^
	46 (32.5; 65.5)	33 (27.5; 44.5)		
Sex (M/F), *n* (%)	19/18 (51.4/48.6)		16/15 (51.6/48.4)	0.981 ^Ϯ^
	13/12 (52/48)	6/6 (50/50)		
BMI (average ± SD)	21.97 ± 1.5		23.08 ± 2.2	0.259 ^#^
	22.61 ± 1.95	21.97 ± 1.5		
Disease activity score	Mayo score 3 (1; 7)	CDAI score 146.5 (52.5; 276.5)		
Active disease, *n* (%)	22 (59.5%)		NA	NA
	16 (64% *)	6 (50% *)	NA	0.65 ^Ϯ^

* Percentage according to disease subtype, ^§^ Kruskal–Wallis test, ^Ϯ^ Chi-square test, ^#^ Student’s *t*-test. UC-ulcerative colitis, CD—Crohn’s disease, BMI—body mass index, CDAI—Crohn’s disease activity index, SD—standard deviation, NA—not applicable.

**Table 2:** Point–biserial correlations between magnesium hair concentration and PSQI components.

PSQI Component	t	df	*p*	CI
Global	−1.05	35	0.3	−0.472, 0.158
Component 1—subjective sleep quality	−1.212	35	0.234	−0.493, 0.132
Component 2—sleep latency	−2.681	35	0.011	−0.65, −0.102
Component 3—Sleep duration	−2.285	35	0.028	−0.613, −0.041
Component 4—Habitual sleep efficiency	−0.706	35	0.485	−0.426, 0.214
Component 5—sleep disturbances	0.644	35	0.524	−0.224, 0.418
Component 6—Use of sleeping medication	−0.942	35	0.353	−0.224, 0.418
Component 7—Daytime dysfunction	−0.725	35	0.474	−0.429, 0.211

CI = confidence interval. PSQI: Pittsburgh Sleep Quality Index.

## Data Availability

The data presented in this study are available on request from the corresponding author.
